# Copy number variant analysis for syndromic congenital heart disease in the Chinese population

**DOI:** 10.1186/s40246-022-00426-8

**Published:** 2022-10-31

**Authors:** Ping Li, Weicheng Chen, Mengru Li, Zhengshan Zhao, Zhiyu Feng, Han Gao, Meijiao Suo, Ziqing Xu, Guixiang Tian, Feizhen Wu, Sheng Wei, Guoying Huang

**Affiliations:** 1 Pediatric Heart Center, Children’s Hospital of Fudan University, No. 399 Wanyuan Road, Shanghai, 201102 People’s Republic of China; 2 Shanghai Key Laboratory of Birth Defects, Shanghai, 201102 People’s Republic of China; 3 Laboratory of Epigenetics, Institutes of Biomedical Sciences, Fudan University, Shanghai, 200032 People’s Republic of China

**Keywords:** Copy number variant, Syndromic congenital heart disease, Chromosomal microarray analysis, Candidate gene

## Abstract

**Background:**

Syndromic congenital heart disease (CHD) is among the most severe conditions in the pediatric population. Copy number variant (CNV) is an important cause of syndromic CHD, but few studies focused on CNVs related to these patients in China. The present study aimed to identify pathogenic CNVs associated with syndromic CHD in the Chinese population.

**Methods:**

A total of 109 sporadic patients with syndromic CHD were applied chromosomal microarray analysis (CMA). Phenotype spectrum of pathogenic or likely pathogenic CNVs was analyzed. CHD-related genes were prioritized from genes within pathogenic or likely pathogenic CNVs by VarElect, OVA, AMELIE, and ToppGene.

**Results:**

Using CMA, we identified 43 candidate CNVs in 37/109 patients. After filtering CNVs present in the general population, 29 pathogenic/likely pathogenic CNVs in 24 patients were identified. The diagnostic yield of CMA for pathogenic/likely pathogenic CNVs was 23.1% (24/104), excluding 5 cases with aneuploidies or gross chromosomal aberrations. The overlapping analysis of CHD-related gene lists from different prioritization tools highlighted 16 CHD candidate genes.

**Conclusion:**

As the first study focused on CNVs in syndromic CHD from the Chinese population, this study reveals the importance of CMA in exploring the genetic etiology of syndromic CHD and expands our understanding of these complex diseases. The bioinformatic analysis of candidate genes suggests several CHD-related genes for further functional research.

**Supplementary Information:**

The online version contains supplementary material available at 10.1186/s40246-022-00426-8.

## Introduction

Syndromic congenital heart disease (CHD) accounts for approximately 20% of all patients with CHD [1], placing a heavy burden on the healthcare system. Extracardiac malformations in patients with CHD may influence their perioperative management, cardiac outcome, and mortality [2]. Chromosomal aberrations are common pathogenic causes in patients with syndromic CHD. Aneuploidies, including trisomy 21, trisomy 18, trisomy 13, and Turner syndrome, account for approximately 14% of all genetic causes of syndromic CHD. Copy number variants (CNVs), including 22q11 deletion, 1p36 deletion, 7q11.23 deletion, and other CNVs account for approximately 20% [1].

CNVs are crucial structural variants in the human genome caused by a deletion or duplication of genomic segments [3]. Identification of CNVs is a concern for children with congenital structural anomalies or multiple developmental disabilities. Chromosomal microarray analysis (CMA), including array comparative genomic hybridization (Array-CGH) and single-nucleotide polymorphism array, can identify chromosomal aberrations in an additional 12–15% of affected children compared with karyotyping [4]. Therefore, the American College of Medical Genetics (ACMG) standards and guidelines recommend CMA as a first-tier diagnostic strategy for patients with intellectual disabilities, autism spectrum disorders, and other multiple congenital anomalies [5]. In 2007, Thienpont et al. evaluated chromosomal aberration in 60 cases of syndromic CHD from Belgium with Array-CGH. They found 16.6% (10/60) of patients carrying causal CNVs [6]. Later, several studies evaluated the diagnostic yield of CMA from different countries or ethnic backgrounds [6–15]. Among these studies, the two cohorts with the largest sample sizes were the BCM1 (104 Hispanic/Latino Americans and 99 non-Hispanic patients of European descent) and BCH (260 cases from American) cohorts [9, 16]. The diagnostic yields of CMA in the two cohorts were 32.5% (66/203) and 18.1% (47/260), respectively. Although research on the relationship between CNVs and syndromic CHD is ongoing, no previous cohort studies have specifically reported CNVs in syndromic CHD from the Chinese population. In this study, we aimed to investigate the CNVs in syndromic CHD from the Chinese population and prioritize critical candidate genes.

## Methods

### Subjects and samples

A group of 109 sporadic patients with syndromic CHDs was recruited for this study. All patients were diagnosed with CHD and extracardiac malformations. Diagnoses were confirmed via imaging, clinical, and laboratory inspections. Patent ductus arteriosus (PDA) in children under one-year-old and patent foramen ovale were excluded. Peripheral blood samples were collected at the outpatient clinic and the inpatient ward of the Cardiothoracic Surgery Department. The Children’s Hospital of Fudan University ethics committee approved the study. The individuals’ parents signed the informed consent for the study, which follows the principles of the Declaration of Helsinki.

### Chromosomal microarray analysis

**Fig. 1:**
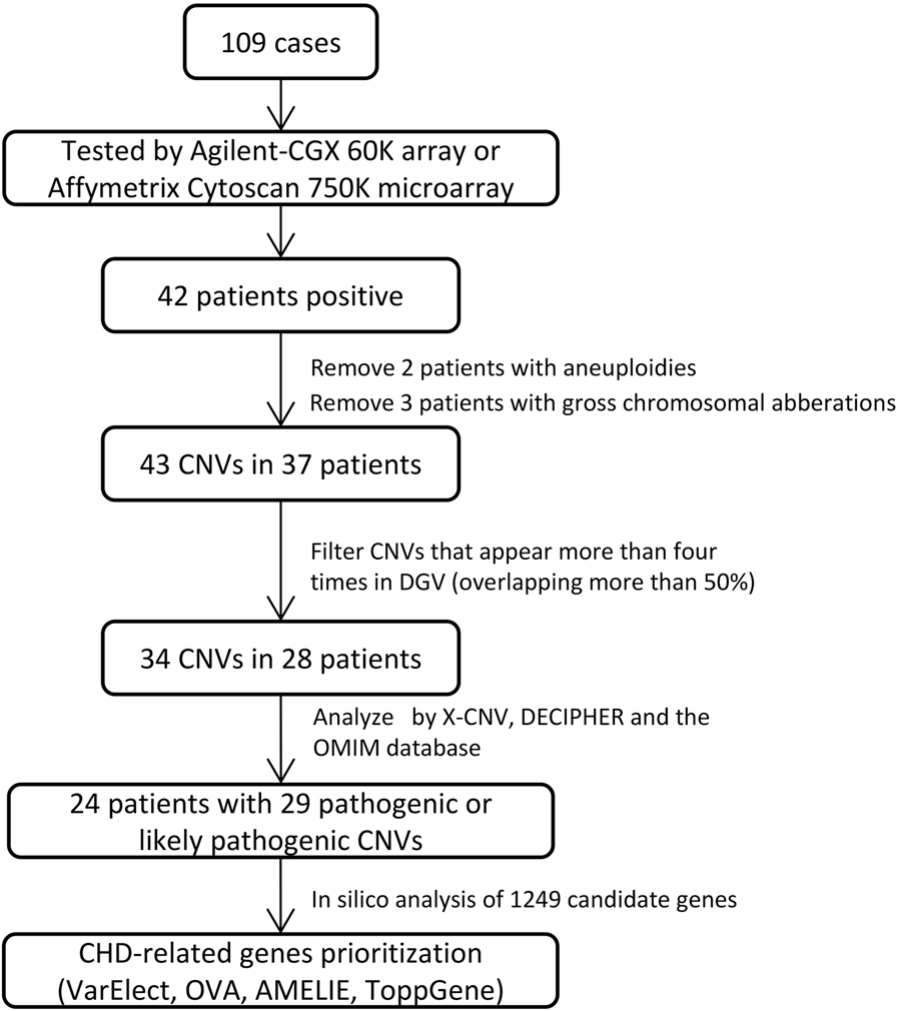
Flowchart of this study

Genomic DNA was extracted from peripheral blood using a QIAamp DNA Blood Kit (Qiagen). After enzyme cutting, labeling, hybridization, and purification, genomic DNA was submitted for CMA using the Agilent-CGX 60 K array or Affymetrix CytoScan 750 K microarray platforms. Details of the microarray technology and variant calling have been reported previously [17, 18]. Detected CNVs meeting the following criteria were excluded for further analysis: 1) gross chromosomal aberrations, including the size of CNV over 30 Mb; 2) CNVs with more than four occurrences in the Database of Genomic Variants (overlapping more than 50%). The remaining CNVs were interpreted using X-CNV (http://119.3.41.228/XCNV/index.php) [19], the DatabasE of genomiC varIation and Phenotype in Humans using Ensembl Resources (DECIPHER, https://www.deciphergenomics.org/) [20], and the Online Mendelian Inheritance in Man database (OMIM, https://www.omim.org/) [21]. CNVs were defined as pathogenic or likely pathogenic if any of the three web tools indicated pathogenicity or likely pathogenicity. X-CNV is a web tool to predict the pathogenicity of CNVs by integrating more than 30 informative features such as allele frequency, CNV length, CNV type, and some deleterious scores. In the development of X-CNV, Zhang et al. [22] reprocessed high-quality CNV data from multiple sources, including dbVar, DECIPHER, ClinGen, and the DGV databases. According to the meta-voting prediction (MVP) score generated by X-CNV, CNVs were divided into five categories: pathogenic, likely pathogenic, uncertain, likely benign, and benign. CNVs overlapped with regions interpreted by the DECIPHER database were defined as the corresponding pathogenicity. As for the OMIM database, CNVs were considered pathogenic when they presented genes associated with diseases. Moreover, CNVs were considered likely pathogenic when they presented genes associated with phenotypic alterations in the OMIM database [23]. The genome reference of X-CNV was GRCh37/hg19. When tracking CNVs in the DECIPHER database (GRCh38), NCBI-remap (https://www.ncbi.nlm.nih.gov/genome/tools/remap) was used for the genome conversion. The flowchart of this study is shown in Fig. 1.

### Phenotype spectrum of syndromic CHD with pathogenic or likely pathogenic CNVs in this study

To further explore the distribution of the phenotype spectrum in CNV patients with syndromic CHD, the cardiac and non-cardiac phenotypes were analyzed. Each region of pathogenic or likely pathogenic CNVs in this study was searched in the DECIPHER database, and all overlapping CNVs were extracted for phenotype analysis.

### Gene prioritization to identify CHD candidate genes

We developed a gene prioritization process to identify CHD candidate genes by integrating various web tools and databases (Additional file 1: Table S1), including phenotype-driven web tools (VarElect [24], OVA [25], and AMELIE [26]) and ToppGene [27]. For ToppGene, the training gene set was generated from RDDC (https://rddc.tsinghua-gd.org/), Phenopedia [28] (https://phgkb.cdc.gov/PHGKB/startPagePhenoPedia.action), and DisGeNET [29] (https://www.disgenet.org/), which contain genes related to CHD based on research articles and database mining (Additional file 2: Table S2). 1354 genes were finally defined as the training gene set [28, 29]. The 1249 input genes for all web tools were defined from protein-coding genes within pathogenic and likely pathogenic CNVs in this study by the UCSC genome browser (Human GRCh37/hg19) [30]. Then, we performed pathway analysis (Additional file 3: Additional methods) and analyzed the expression profile of the overlapping prioritized genes between four tools during murine cardiogenesis.

### Statistical analysis

Statistical analyses were performed using GraphPad Prism (version 8.0).

## Results

### Clinical features and chromosomal imbalances in patients with syndromic CHD

A total of 109 patients with syndromic CHD underwent CMA analysis. The cases included 70 males and 39 females, with a mean age of 1.7 years (0–9.6 years). Among all cardiac phenotypes in this cohort (Table 1), septal defects were observed in 66.1% (72/109) of the patients, compound conotruncal defects in 10.1% (11/109), and obstruction of left ventricular outflow tract in 7.3% (8/109). The remaining 16.5% (18/109) of the patients presented septal defects with abnormities of valves, isolated abnormities of valves, isolated conotruncal defects, heterotaxy syndrome, and other cardiac defects. The main extracardiac comorbidities of all patients were neurodevelopmental disorders (37/109, 33.9%), craniofacial defects (13/109, 11.9%), genitourinary defects (12/109, 11.0%), digestive system defects (11/109, 10.1%), and musculoskeletal disorders (11/109, 10.1%).

**Table 1:** CHD and extracardiac phenotypes in patients with syndromic CHD

CHD phenotypes	Total N (%)	Neurodevelopmental disorders n (%)	Genitourinary defects n (%)	Craniofacial defects n (%)	Digestive system defects n (%)	Limbs defects n (%)	Musculoskeletal disorders n (%)	Respiratory system defects n (%)	Endocrine system disorders n (%)	Others n (%)
A. Septal defects	72 (66.1)	30 (41.7)	10 (13.9)	7 (9.7)	7 (9.7)	4 (5.6)	7 (9.7)	2 (2.8)	4 (5.6)	1 (1.4)
B. Isolated abnormities of valves	3 (2.8)	0 (0.0)	1 (33.3)	0 (0.0)	0 (0.0)	0 (0.0)	0 (0.0)	0 (0.0)	1 (33.3)	1 (33.3)
C. Septal defects with abnormities of valves	5 (4.6)	2 (40.0)	0 (0.0)	2 (40.0)	0 (0.0)	1 (20.0)	0 (0.0)	0 (0.0)	0 (0.0)	0 (0.0)
D. Obstruction of left ventricular outflow tract	8 (7.3)	2 (25.0)	1 (12.5)	1 (12.5)	2 (25)	0 (0.0)	0 (0.0)	1 (12.5)	1 (12.5)	0 (0.0)
E. Isolated conotruncal defects	3 (2.8)	0 (0.0)	0 (0.0)	0 (0.0)	1 (33.3)	0 (0.0)	1 (33.3)	0 (0.0)	0 (0.0)	1 (33.3)
F. Compound conotruncal defects	11 (10.1)	3 (27.3)	0 (0.0)	2 (18.2)	1 (9.1)	0 (0.0)	3 (27.3)	0 (0.0)	0 (0.0)	2 (18.2)
G. Heterotaxy syndrome	1 (0.9)	0 (0.0)	0 (0.0)	1 (100.0)	0 (0.0)	0 (0.0)	0 (0.0)	0 (0.0)	0 (0.0)	0 (0.0)
H. Others	6 (5.5)	0 (0.0)	0 (0.0)	0 (0.0)	0 (0.0)	1 (16.7)	0 (0.0)	4 (66.7)	0 (0.0)	1 (16.7)
Total	109	37 (33.9)	12 (11.0)	13 (11.9)	11 (10.1)	6 (5.5)	11 (10.1)	7 (6.4)	6 (5.5)	6 (5.5)

This categorization was adapted from the classification system established by Geng [9] et al

Each category includes the following CHD phenotypes: A: ASD, VSD, and AVSD; B: TR, MR, PS, MS, bicuspid aortic valve, and supramitral stenosing ring; C: ASD/VSD with abnormities of valves; D: AS and CoA; E: TOF, DORV, TGA; F: TOF/DORV/TGA/PA with other heart defects; G: heterotaxy syndrome; and H: PAPVC, PAS, SV, PDA, truncus arteriosus, hypoplastic left heart syndrome, aortic regurgitation, etc.

For patients with extracardiac comorbidities involving multiple systems, only the main or the most severe extracardiac comorbidity was considered in this table

We identified two patients with aneuploidies: one with trisomy 19 and one with trisomy 21 (Additional file 4: Table S3, cases 3 and 51). Three patients with gross chromosomal aberrations were found (Additional file 4: Table S3, cases 41, 74, and 95). The duplication of 3q26.1-q29 (34.8 Mb) existed in case 41. Case 74 carried the duplications of 18 CNVs, including 2q31.2-q35 (37.2 Mb), 3p26.3-p26.1 (3.6 Mb), 3p26.1-p25.3 (5.6 Mb), 3q28-q29 (6.4 Mb), 4p16.1-p15.32 (6.5 Mb), 4p15.1-p14 (5.6 Mb), 4q26-q31.3 (36.7 Mb), 5p15.1-p14.1 (11.0 Mb), 6q16.3-q21 (3.8 Mb), 8q21.13-q23.1 (26.2 Mb), 8q23.2-q23.3 (4.7 Mb), 10q22.3-q25.2 (32.7 Mb), 11p14.3-p11.2 (21.8 Mb), 11q12.1-q13.5 (20.1 Mb), 13q33.3-q34 (1.7 Mb), 17q22-q24.1 (7.9 Mb), 18q22.3-q23 (7.3 Mb), and 21q21.2-q22.11 (8.6 Mb). The duplications of 2p25.3-p11.2 (85.0 Mb), 2q11.1-q37.3 (143.8 Mb), and 20q11.21-q13.12 (12.8 Mb) were present in case 95. Apart from gross chromosomal aberrations, 37 patients carried 43 CNVs in this study. Five previously reported syndromes involving complex congenital malformations were also present in this cohort, including 1p36 microdeletion syndrome (case 11), DiGeorge syndrome (cases 32 and 66), Miller–Dieker syndrome (case 76), Cri du Chat syndrome (case 99), and Smith–Magenis syndrome (case 107). The overall rate of chromosomal imbalances in patients with syndromic CHD was 38.5% (42/109).

### Pathogenic CNVs in patients with syndromic CHD

CNVs that appear more than four times in DGV (overlapping more than 50%) were regarded as common CNVs, as reported previously [12]. To find rare pathogenic CNVs, we filtered common CNVs and analyzed the remaining 34 CNVs by X-CNV, DECIPHER, and the OMIM database. 29 pathogenic or likely pathogenic CNVs in 24 patients were finally identified (Table 2). 22q11.2 was the only recurrent CNV. The 29 CNVs contained 1249 protein-coding genes. WEB-based GEne SeT AnaLysis Toolkit (WebGestalt, http://www.webgestalt.org/) [31] was used for the gene ontology annotation of these genes (Additional file 5: Fig. S1).

**Table 2:** Pathogenic or likely pathogenic CNVs in 24 patients with syndromic CHD

Patient No	Sex	Age	CNVs: Region and size	Range	X-CNV MVP Score	Decipher	OMIM	CHD-related genes	Syndrome (Decipher)	CHD	Extracardiac malformations
5	M	7 m	Dup 8q24.21-q24.3 (17.7 Mb)	Chr8:128,538,700–146,262,124	Pathogenic: 0.862	Pathogenic	Pathogenic	*AGO2*, *CYP11B2*^a^, *FOXH1*^a^, *KCNK9*, *PTK2*^a^, *SLURP1*, *CYP11B1*^a^, *NDRG1*^a^, *SLC39A4*^a^	/	ASD	Mental retardation, cryptorchidism
			Del 13q33.1-q34 (11.9 Mb)	Chr13:103,226,118–115,091,802	Pathogenic: 0.840	Pathogenic	Pathogenic	*COL4A1*^a^, *EFNB2*^a^, *F10*^a^, *IRS2*^a^, *COL4A2*^a^, *GAS6*^a^	/		
11	M	1y2m	Del 1p36.33 (1.3 Mb)	Chr1:849,466–2,174,235	Pathogenic: 0.840	Pathogenic	Pathogenic	*DVL1*, *GNB1*, *AGRN*^a^, *SKI*^a^	1p36 microdeletion syndrome	VSD	Mental retardation
12	M	7 m	Del 2p16.3 (0.03 Mb)	Chr2:50,203,462–50,232,894	Benign: 0.123	NM	Pathogenic	*NRXN1*	/	TOF, RAA, abnormal origin of right coronary artery	Skewed mouth, left ear deformity, hydrocele
17	M	4y2m	Del 3q25.33-q26.1 (0.2 Mb)	Chr3:160,526,260–160,738,728	Likely pathogenic: 0.527	Likely benign	NM	/	/	DORV, TGA, VSD, PS, PDA, abnormal right coronary artery branch	Hydrocele, genu valgum
32	M	6 m	Del 22q11.21 (2.5 Mb)	Chr22:18,921,359–21,460,595	Pathogenic: 0.840	Pathogenic	Pathogenic	*COMT*^a^, *CRKL*^a^, *SLC25A1*, *SNAP29*, *TBX1*^a^, *TXNRD2*^a^, *LZTR1*^a^	22q11.2 deletion syndrome (DiGeorge syndrome)	TOF, RAA	Athymism, immunodeficiency
33	M	3y9m	Del 2q13 (1.7 Mb)	Chr2:111,398,336–113,101,220	Likely pathogenic: 0.527	Uncertain	Pathogenic	*MERTK*^a^, *BCL2L11*^a^	/	ASD	Funnel chest
34	M	4y7m	Dup Xp22.31 (0.5 Mb)	ChrX:6,836,073–7,347,549	Likely benign: 0.154	Likely pathogenic	NM	*STS*	/	VSD	Cerebral dysplasia, mental retardation, multiple deformities of vertebrae
38	F	3 m	Del 17q25.3 (0.8 Mb)	Chr17:79,344,285–80,192,099	Likely pathogenic: 0.527	NM	Pathogenic	*ARHGDIA*^a^, *PYCR1*^a^	/	VSD, ASD, left aortic arch with right subclavian artery voyage	hydrocephalus, arachnoid cyst, congenital hip dysplasia
43	F	4 m	Del 5q35.3 (0.6 Mb)	Chr5:180,069,797–180,686,444	Likely pathogenic: 0.527	NM	Pathogenic	*FLT4*^a^	/	VSD, abnormal right ventricular muscle bundle	Transverse facial cleft
44	M	6 m	Del 1q43-q44 (5.5 Mb)	Chr1:243,545,786–249,208,146	Likely pathogenic: 0.527	Pathogenic	Pathogenic	*AKT3*, *HNRNPU*^a^, *NLRP3*, *SDCCAG8*	/	ASD	Cerebral dysplasia
53	M	2 m	Del 6q14.1-q16.1 (11.0 Mb)	Chr6:83,849,802–95,155,354	Likely pathogenic: 0.544	Pathogenic	Pathogenic	*MAP3K7*^a^, *NT5E*^a^	/	VSD, ASD	Cryptorchidism, mental retardation
60	M	2 m	Dup 15q11.2 (1.0 Mb)	Chr15:24,050,216–25,107,421	Uncertain: 0.187	Uncertain	Likely pathogenic	/	/	Solitary dextrocardia, bilateral right atrium heterogeneity, ventricular reversal, complete AVSD, anatomical DORV with TGA, PVS	Heterotaxy, cleft lip, cleft palate
66	M	2 m	Del 22q11.21 (2.5 MB)	Chr22:18,919,528–21,460,595	Pathogenic: 0.840	Pathogenic	Pathogenic	*COMT*^a^, *CRKL*^a^, *SLC25A1*, *SNAP29*, *TBX1*^a^, *TXNRD2*^a^, *LZTR1*^a^	22q11.2 deletion syndrome (DiGeorge syndrome)	VSD	Abnormal facial features, narrow glottis
**75**	F	4 m	Del 11q23.3-q25 (13.5 Mb)	Chr11:121,057,903–134,928,850	Pathogenic: 0.840	Pathogenic	Pathogenic	*CDON*^a^, *CHEK1*, *ETS1*, *HYLS1*^a^, *KCNJ5*^a^, *SC5D*^a^, *SCN3B*^a^, *JAM3*^a^	/	DORV, CoA	Familial exudative vitreoretinopathy, horseshoe kidney, foot deformity, skull deformity
**76**	M	1y1m	Del 17p13.3 (0.2 Mb)	Chr17:226,043–438,909	Likely pathogenic: 0.544	NM	Pathogenic	/	Miller–Dieker syndrome	VSD	Mental retardation
			Dup 18p11.31-p11.23 (0.5 Mb)	Chr18:7,086,919–7,605,032	Uncertain: 0.282	NM	Pathogenic	/			
**77**	F	1y1m	Dup 7q34-q36.3 (19.1 Mb)	Chr7:139,520,175–159,123,167	Pathogenic: 0.862	Pathogenic	Pathogenic	*BRAF*^a^, *CNTNAP2*, *DNAJB6*, *DPP6*, *EZH2*^a^, *KCNH2*^a^, *MNX1*, *PRKAG2*^a^, *RHEB*^a^, *SHH*^a^, *PRSS1*^a^	/	VSD	Hydrocephaly
**96**	F	5y10m	Dup Xq26.2 (0.1 Mb)	ChrX:133,025,264–133,159,421	Likely benign: 0.141	NM	Pathogenic	*GPC3*^a^	/	ASD	Single transverse palmar crease, mental retardation
**99**	F	11 m	Del 5p15.33-p15.31 (7.8 Mb)	Chr5:113,576–8,101,272	Pathogenic: 0.840	Pathogenic	Pathogenic	/	Cri du Chat Syndrome (5p deletion)	VSD	mental retardation, motor retardation
			Dup 5p15.31-p14.1 (19.1 Mb)	Chr5:8,115,306–27,645,325	Pathogenic: 0.862	Pathogenic	Pathogenic	*DNAH5*^a^, *MTRR*	/		
**102**	M	3y	Dup 3p26.3-p24.2 (25.7 Mb)	Chr3:105,511–25,830,553	Pathogenic: 0.862	Pathogenic	Pathogenic	*CAV3*^a^, *COLQ*^a^, *CRELD1*^a^, *RAB5A*^a^, *RAF1*^a^, *RARB*^a^, *RPL15*, *SLC6A6*^a^, *SUMF1*^a^, *THRB*^a^, *TMEM43*, *VHL*^a^, *XPC*, *CRBN*^a^, *ITPR1*, *PPARG*^a^, *WNT7A*^a^	/	VSD, ASD	Hypothyroidism, developmental delay
**103**	F	1y11m	Del 9p24.3-p22.3 (14.5 Mb)	Chr9:204,149–14,724,068	Pathogenic: 0.840	Pathogenic	Pathogenic	*GLDC*^a^, *JAK2*^a^, *KANK1*, *MPDZ*^a^, *VLDLR*^a^	/	ASD	Cleft palate, mental retardation
			Dup 9p22.3 (1.6 MB)	Chr9:14,762,293–16,349,620	Likely benign: 0.141	Likely pathogenic	Pathogenic	/	/		
			Dup 10p15.1-p14(1 MB)	Chr10:6,547,235–7,565,244	Likely benign: 0.141	Uncertain	NM	/	/		
**104**	M	1y	Del 16q21-q22.1 (1.4 Mb)	Chr16:66,280,192–67,654,588	Likely pathogenic: 0.527	NM	Likely pathogenic	*CTCF*, *HSD11B2*^a^, *HSF4*, *CBFB*^a^, *NOL3*^a^	/	VSD, ASD, PDA	Cryptorchidism, oblique inguinal hernia, hemangioma
**107**	M	11 m	Del 17p11.2 (3.7 Mb)	Chr17:16,603,146–20,274,157	Pathogenic: 0.840	Pathogenic	Pathogenic	*B9D1*^a^, *FLCN*^a^, *TNFRSF13B*, *MAPK7*^a^	Smith–Magenis syndrome	VSD	Developmental disorder of speech and language, motor retardation
**108**	F	6y	Del 3p22.2 (0.6 Mb)	Chr3:38,455,532–39,035,153	Pathogenic: 0.854	Pathogenic	Pathogenic	*ACVR2B*^a^, *SCN5A*^a^	/	ASD, PVS	Genital tract malformation
**109**	F	6y	Dup 6p25.3-p22.2 (24.7 Mb)	Chr6:390,212–25,638,706	Pathogenic: 0.862	Likely pathogenic	Pathogenic	*CAP2*^a^, *EDN1*^a^, *FOXC1*^a^, *SOX4*^a^, *TFAP2A*^a^	/	PDA	Rhinostenosis
			Del 15q26.3 (1.0 Mb)	Chr15:101,341,696–102,391,143	Pathogenic: 0.840	Uncertain	Likely pathogenic	*CHSY1*	/		

CHD-related genes were identified if the genes were prioritized in at least three tools of VarElect, OVA, AMELIE, and ToppGene

NM: not mentioned

^a^ MGI showed cardiovascular system phenotypes in the targeted homozygous null allele mice

**Fig. 2:**
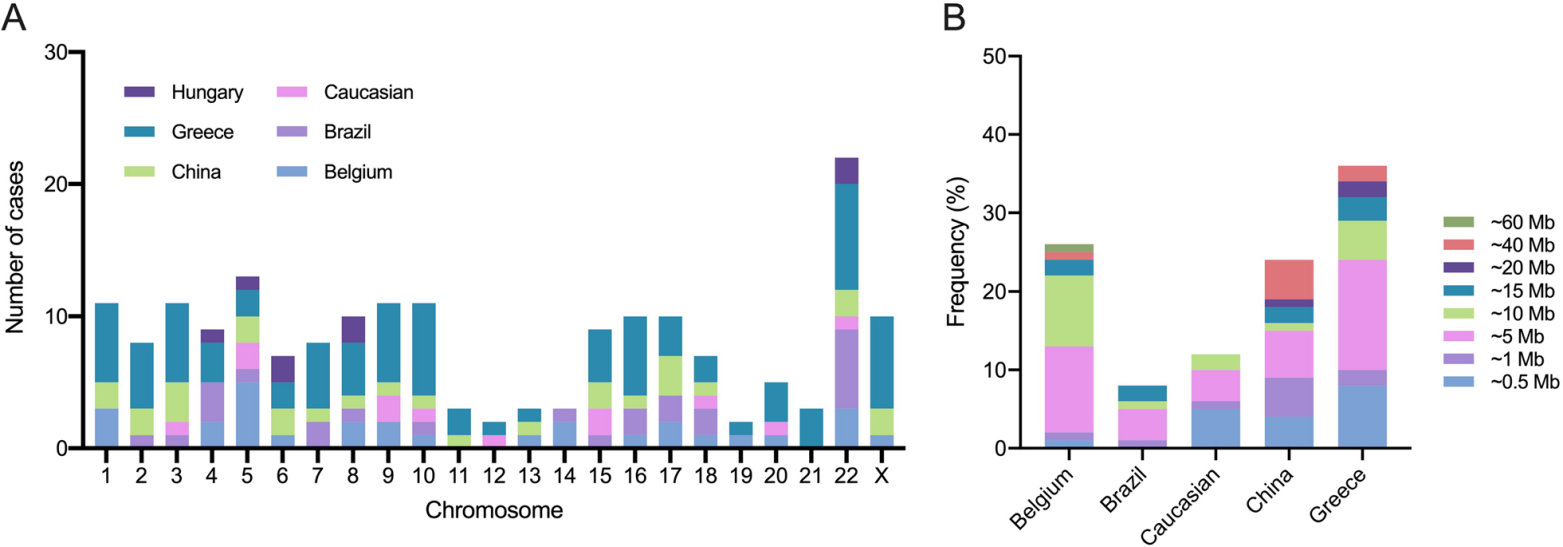
Characteristics of CNVs in studies of syndromic CHD from different countries or ethnicities. **A** Distribution of CNV on different chromosomes. **B** Distribution of CNV sizes

Then, we compared the characteristics of CNVs in studies of syndromic CHD from different countries or ethnicities (Table 3). Detailed information on CNVs for patients from Hungary [10], Greece [7], Brazil [12, 14], Belgium [6, 8], and the Caucasian population [15] was provided. As shown in Fig. 2A, among cases from Greece and Brazil, CNVs were mainly in chromosome 22. 22q11.2 was the most frequent region (Additional file 6: Table S4). In China, CNVs were more evenly distributed across chromosomes. We also compared the sizes of CNVs per individual (Fig. 2B). The CNV sizes of patients from Hungary were not provided, so we excluded these patients. Similar to patients from other countries or ethnicities, this study's most common size of CNVs in syndromic CHD was 1–5 Mb. In addition, we found a higher percentage of 20–40 Mb CNV sizes in Chinese patients.

**Table 3:** Diagnostic yield of CMA in studies of syndromic CHD from different countries or ethnicities

No	Country or ethnicity	Year	Ranging size	Platform	Criteria for pathogenic/likely pathogenic or causal CNVs	Diagnostic yield of CMA	CHD (top 3)*	Extracardiac malformations (top 3)
1 [6]	Belgium	2007	0.15–14 Mb	1 Mb BAC/PAC clone set	meet any of the following criteria: 1. contain genes known to cause CHD or another dominant monogenic disease through a dosage effect 2. other individuals with the same CNV and phenotype 3. de novo 4. over 20 affected genes	10/60 (16.6%)	E (40%), A (20%), D (20%)	Craniofacial defects (60.0%), neurodevelopmental disorders (50.0%), genitourinary defects (20.0%)
2 [11]	12 European-Americans, 7 Hispanics, and 1 Asian	2008	0.07–14.1 Mb	Nimblegen 385 K CGH	absent in DGV; contain known or hypothetical genes	5/20 (25%)	A (40%), E (40%), D (20%)	Neurodevelopmental disorders (100%), genitourinary defects (20%), limbs defects (20%)
3 [8]	Belgium	2010	0.22–45.09 Mb	1 Mb BAC/PAC clone set	meet any of the following criteria: 1. previously reported in DECIPHER, ECARUCA, CHDWiki, or OMIM 2. CNV causes a mutation in a gene known to cause an autosomal recessive disorder similar to the patient’s phenotype and both alleles are mutated	16/90 (17.8%)	A (56.3%), H (18.8%), C (12.5%)	Craniofacial defects (93.8%), neurodevelopmental disorders (81.3%), limbs defects (62.5%)
4 [15]	Caucasian	2011	0.23–9.6 Mb	Affymetrix GeneChip 100 K array	absent in large control datasets; contain recognized genes	12/58 (20.7%)	A (66.7%), F (16.7%), H (16.7%)	Craniofacial defects (75%), limbs defects (50%), musculoskeletal disorders (50%)
5 [16]	104 Hispanic/Latino Americans and 99 non-Hispanic patients of European descent	2012	0.05–36 Mb	Agilent customized 105 K CGH array	> 50 kb; had DGV overlap of ≤ 75%; contained at least one known gene and absent in the controls	70/203 (34.5%)	not specified	Not specified
6 [7]	Greece	2013	0.08–19.01 Mb	Agilent 244 K CGH array or Agilent 4 × 180 K SNP + CGH array	contain significant candidate genes relating to CHD	37/55 (67.3%)	A (62.2%), H (18.9%), B (16.2%)	Neurodevelopmental disorders (70.3%), other ECMs not specified
7 [9]	America	2014	Not specified	Agilent 244 K CGH array or Agilent 4 × 180 K SNP + CGH array	ACMG standards and guideline	47/260 (18.1%)	not specified	Not specified
8 [12]	Brazil	2017	0.60–17.4 Mb	Agilent Human Genome G3 SurePrint 8 × 60 K microarray or Affymetrix CytoScan HD chip	≥ 300 kb; relevant CNVs searched in DGV, DECIPHER, and OMIM	8/78 (10.3%)	F (62.5%), H (25.0%), B (12.5%)	Craniofacial defects (100%), Neurodevelopmental disorders (87.5%), musculoskeletal disorders (62.5%)
9 [14]	Brazil	2017	Not specified	multiplex ligation-dependent probe amplification(MLPA) or Affymetrix CytoScan 750 K array	described in DECIPHER database or in PubMed	12/47 (25.5%)	A (58.3%), D (33.3%), C (8.3%)	Craniofacial defects (75%), neurodevelopmental disorders (66.7%), genitourinary defects (8.3%)
10 [13]	Saudi Arabia	2018	0.01–11,530 Mb	Agilent array-CGH 2 × 400 K or Agilent CGH/SNP 2 × 400 K microarray	reported in public database and literatures being associated with known disease and likely to be clinically significant	15/73 (20.5%)	not specified	Not specified
11 [10]	Hungary	2019	0.004–34.58 Mb	Affymetrix CytoScan 750 K array	ACMG standards and guideline	7/33 (21.2%)	E (42.9%), A (28.6%), D (14.3%)	Not specified
12	China (our study)	2022	0.2–25.7 Mb	Agilent-CGX 60 K array or Affymetrix CytoScan 750 K array	X-CNV, DECIPHER, and OMIM	24/104 (23.1%)	A (66.1%), F (10.1%), D (7.3%)	Neurodevelopmental disorders (33.9%), craniofacial defects (11.9%), genitourinary defects (11.0%)

^*^The A-H classification of CHD is described in Table 1

### Phenotype spectrum of pathogenic or likely pathogenic CNVs in this study

**Fig. 3:**
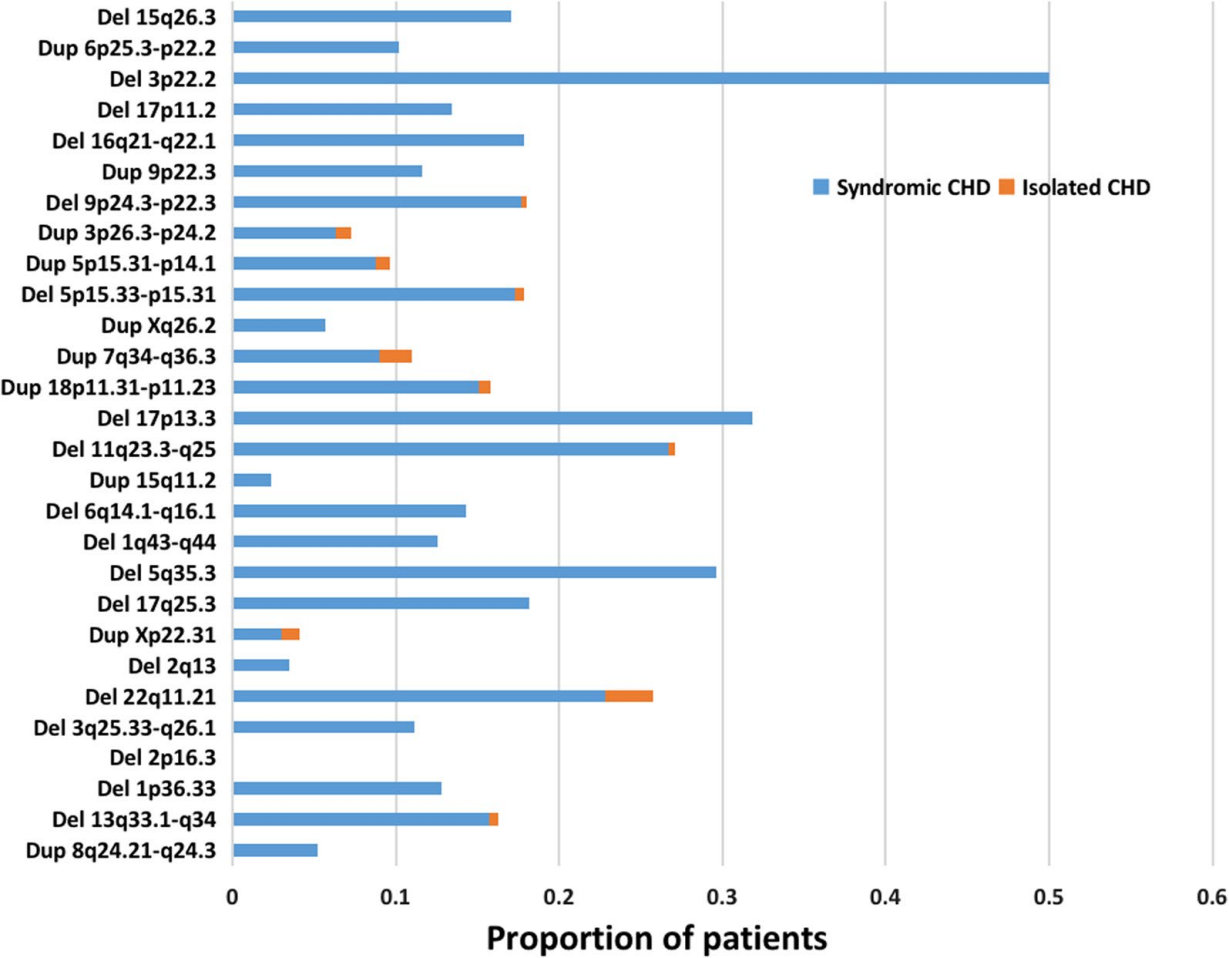
Percentage distribution of patients with syndromic and isolated CHD, across each pathogenic or likely pathogenic CNV type in this study. The percentage was summarized by searching the region of each CNV in DECIPHER database

Syndromic and isolated CHD prevalence in CNV patients from DECIPHER was analyzed. As shown in Fig. 3 and Table 4, ten of all CNVs were both related to syndromic and isolated CHD. The percentage of isolated CHD in each CNV was much lower than syndromic CHD. Then, we analyzed the detailed phenotype spectrum in CNV patients with syndromic and isolated CHD (Table 4). Septal defects and intellectual disabilities were the most common cardiac and non-cardiac phenotypes in CNV patients with syndromic CHD. For isolated CHD, complex conditions were more common, such as tetralogy of Fallot. Differential disease-associated genes (according to OMIM) between isolated CHD from DECIPHER and syndromic CHD in this study are also analyzed in Table 4. These genes may be candidate genes for non-cardiac phenotypes of CNV patients with syndromic CHD.

**Table 4:** The phenotypes of each pathogenic or likely pathogenic CNV in DECIPHER database

No	CNV	Syndromic CHD^*^	Top 5 extracardiac phenotypes of syndromic CHD (*n*)	Top 5 cardiac phenotypes of syndromic CHD (*n*)	Isolated CHD^*^	Phenotypes of isolated CHD	Differential genes between isolated CHD from DECIPHER and syndromic CHD in this study (Disease-associated genes according to OMIM)^#^
1	Dup 8q24.21-q24.3	5.2% (7/134)	Intellectual disability (4), Preauricular skin tag (2), Micrognathia (2), Low-set ears (2), High palate (2)	Ventricular septal defect (3), Tetralogy of Fallot (1), Pulmonic stenosis (1), Abnormal heart morphology (1), Atrial septal defect (1)	0	/	/
2	Del 13q33.1-q34	15.7% (27/172)	Intellectual disability (14), Microcephaly (11), Micrognathia (11), Small for gestational age (11), Low-set ears (9)	Ventricular septal defect (12), Atrial septal defect (8), Abnormality of cardiovascular system morphology (6), Tetralogy of Fallot (3), Pulmonic stenosis (2)	0.6% (1/172)	Tetralogy of Fallot (1)	SLC10A2
3	Del 1p36.33	12.8% (25/195)	Intellectual disability (12), EEG abnormality (8), Microcephaly (7), Frontal bossing (6), Hypotonia (6)	Ventricular septal defect (9), Patent ductus arteriosus (8), Atrial septal defect (6), Tetralogy of Fallot (2), Abnormal heart morphology (1)	0	/	/
4	Del 2p16.3	0 (0/12)	/	/	0	/	/
5	Del 3q25.33-q26.1	11.1% (1/9)	Abnormal esophagus morphology (1), Anosmia (1), Blepharophimosis (1), Choanal atresia (1), Hydrocephalus (1), Hypertelorism (1), Micrognathia (1), Renal hypoplasia (1), Talipes equinovarus (1), Tracheoesophageal fistula (1)	Ventricular septal defect (1)	0	/	/
6	Del 22q11.21	22.8% (170/745)	Intellectual disability (53), Hypocalcemia (46), Aplasia/Hypoplasia of the thymus (44), Micrognathia (44), Abnormal pinna morphology (38)	Ventricular septal defect (85), Abnormality of cardiovascular system morphology (33), Tetralogy of Fallot (30), Atrial septal defect (23), Truncus arteriosus (22)	3.0% (22/745)	Tetralogy of Fallot (7), Abnormality of cardiovascular system morphology (5), Ventricular septal defect (4), Pulmonic stenosis (3), Abnormal ventricular septum morphology (2)	ADA2, ATP6V1E1, IL17RA, PEX26, PRODH, TUBA8, USP18, BCR, CHCHD10, CRYBB2, CRYBB3, GGT1, IGLL1, MAPK1, MIF, SMARCB1, SPECC1L, UPB1
7	Del 2q13	3.4% (2/58)	Feeding difficulties in infancy (1), Hypotonia (1), Microcephaly (1), Proportionate short stature (1), Talipes (1)	Atrioventricular canal defect (1),Ventricular septal defect (1), Tetralogy of Fallot (1)	0	/	/
8	Dup Xp22.31	3.0% (8/269)	Conductive hearing impairment (8), Intellectual disability (8), Protruding ear (8)	Ventricular septal defect (8)	1.1% (3/269)	Atrial septal defect (2), Abnormal heart morphology (1)	STS
9	Del 17q25.3	18.2% (2/11)	Camptodactyly of finger (2), Downslanted palpebral fissures (2), Generalized hirsutism (2), High palate (2), Micrognathia (2)	Abnormality of cardiovascular system morphology (2), Hypoplastic left heart (1), Mitral stenosis (1)	0	/	/
10	Del 5q35.3	29.6% (8/27)	Feeding difficulties in infancy (4), Abnormal pinna morphology (3), Anteverted nares (3), Depressed nasal bridge (3), Intellectual disability (3)	Patent ductus arteriosus (4), Atrial septal defect (2), Aortic regurgitation (1), Atrioventricular canal defect (1), Pulmonic stenosis (1)	0	/	/
11	Del 1q43-q44	12.6% (25/199)	Intellectual disability (14), Micrognathia (13), Hypertelorism (8), Low-set ears (8), Depressed nasal bridge (7)	Ventricular septal defect (13), Abnormality of cardiovascular system morphology (3), Pulmonic stenosis (3), Abnormal heart morphology (2), Atrial septal defect (2)	0	/	/
12	Del 6q14.1-q16.1	14.3% (12/84)	Hypotonia (6), Intellectual disability (6), Abnormality of vision (5), Autistic behavior (5), Constipation (5)	Atrial septal defect (4), Patent ductus arteriosus (4), Sinus venosus atrial septal defect (1), Abnormality of the cardiovascular system (1), Ventricular septal defect(1)	0	/	/
13	Dup 15q11.2	2.3% (6/257)	Intellectual disability (3), Delayed speech and language development (2), Hypotonia (2), Overlapping toe (2), Protruding ear (2)	Ventricular septal defect (3), Pulmonic stenosis (2), Tetralogy of Fallot (2), Abnormality of cardiovascular system morphology (1), Atrial septal defect (1)	0	/	/
14	Del 11q23.3-q25	26.7% (66/247)	Intellectual disability (33), Low-set ears (33), Micrognathia (29), Hypertelorism (28), Small for gestational age (23)	Ventricular septal defect (31), Atrial septal defect (16), Patent ductus arteriosus (12), Abnormality of cardiovascular system morphology (11), Hypoplastic left heart (7)	0.4% (1/247)	Hypoplastic left heart (1), Interrupted aortic arch (1)	SC5D, TECTA
15	Del 17p13.3	31.9% (29/91)	Low-set ears (15), Intellectual disability (14), Micrognathia (14), Hypotonia (12), Small for gestational age (12)	Atrial septal defect (12), Patent ductus arteriosus (12), Ventricular septal defect (10), Abnormality of cardiovascular system morphology (7), Tetralogy of Fallot (3)	0	/	/
16	Dup 18p11.31-p11.23	15.1% (21/139)	Intellectual disability (11), Low-set ears (8), Micrognathia (8), Epicanthus (7), High palate (7)	Ventricular septal defect (9), Abnormality of cardiovascular system morphology (5), Atrial septal defect (5), Atrioventricular canal defect (2), Patent ductus arteriosus (2)	0.7% (1/139)	Abnormal heart morphology (1)	LAMA1
17	Dup 7q34-q36.3	9.0% (9/100)	Low-set ears (3), Small for gestational age (3), Delayed speech and language development (2), Downslanted palpebral fissures (2), Global developmental delay (2)	Ventricular septal defect (4), Atrial septal defect (3), Patent ductus arteriosus (2), Abnormal heart morphology (1), Pulmonary artery stenosis (1)	2.0% (2/100)	Abnormal cardiac septum morphology (1), Abnormal aortic valve morphology (1), Tetralogy of Fallot (1)	AGK, BRAF, CLCN1, KEL, PRSS1, PRSS2, SSBP1, TAS2R38, TBXAS1, TRPV6, WEE2, ASB10, CDK5, CNTNAP2, EZH2, GIMAP5, KCNH2, NOBOX, NOS3, TPK1, DNAJB6, DPP6, DYNC2I1, KMT2C, LMBR1, MNX1, NCAPG2, SHH, XRCC2
18	Dup Xq26.2	5.7% (2/35)	Delayed speech and language development (1), Microcephaly (1), Cupped ear (1), Long palpebral fissure (1), Short stature (1)	Tetralogy of Fallot (1), Supravalvular aortic stenosis (1)	0	/	/
19	Del 5p15.33-p15.31	17.3% (31/179)	Micrognathia (17), Intellectual disability (15), Low-set ears (15), Hypertelorism (13), Hypotonia (12)	Ventricular septal defect (12), Atrial septal defect (8), Patent ductus arteriosus (8), Abnormality of cardiovascular system morphology (4), Abnormal heart morphology (3)	0.6% (1/179)	Atrioventricular canal defect (1)	AGXT2, AMACR, ANKH, CCT5, CTNND2, DNAH5, DNAJC21, IL7R, LMBRD2, MARCHF6, NADK2, NPR3, OTULIN, PRLR, RETREG1, SLC1A3, SLC45A2, SPEF2, TARS1, TRIO
20	Dup 5p15.31-p14.1	8.8% (11/125)	Intellectual disability (4), Single transverse palmar crease (3), Iris coloboma (2), Low-set ears (2), Proportionate short stature (2)	Ventricular septal defect (4), Atrial septal defect (3), Patent ductus arteriosus (3), Abnormality of cardiovascular system morphology (1), Mitral regurgitation (1)	0.8% (1/125)	Abnormal heart morphology (1)	CCT5, CTNND2, MARCHF6, ANKH, DNAH5, OTULIN, RETREG1, TRIO
21	Dup 3p26.3-p24.2	6.3% (19/302)	Intellectual disability (6), Micrognathia (5), Delayed speech and language development (4), Downslanted palpebral fissures (4), Low-set ears (4)	Atrial septal defect (8), Ventricular septal defect (6), Abnormal heart morphology (2), Patent ductus arteriosus (2), Abnormal atrioventricular valve morphology (1)	1.0% (3/302)	/	ABHD5, ACOX2, ACVR2B, ACY1, ADAMTS9, AMT, ANO10, APPL1, ARL13B, ARL6, ARPC4, ATG7, ATP2B2, ATXN7, BAP1, BRPF1, BTD, CACNA1D, CACNA2D2, CAV3, CCDC174, CCR2, CCR5, CHMP2B, CIDEC, CISH, CLEC3B, COL7A1, COLQ, CPOX, CRBN, CRELD1, CRTAP, CTNNB1, CX3CR1, DAG1, DALRD3, DAZL, DHX30, DNAH1, DNASE1L3, DOCK3, EOGT, FANCD2, FLNB, FOXP1, FYCO1, GBE1, GHRL, GLB1, GLYCTK, GMPPB, GNAI2, GNAT1, GPD1L, GPX1, GRM7, HESX1, HYAL1, HYAL2, IL17RC, IL17RD, IMPDH2, IQSEC1, ITPR1, JAGN1, KIF15, KLHDC8B, KLHL40, LAMB2, LARS2, LMOD3, LZTFL1, MAPKAPK3, MITF, MLH1, MRPS25, MST1, MST1R, MTMR14, MYD88, MYL3, NBEAL2, NDUFAF3, NEK10, NGLY1, NPRL2, NSUN3, OGG1, P4HTM, PBRM1, PDHB, PLCD1, POC1A, POMGNT2, POU1F1, PPARG, PRKCD, PROK2, PROS1, PTH1R, PTPN23, QARS1, QRICH1, RAF1, RARB, RFT1, RHOA, ROBO2, RPL15, RPSA, SATB1, SCN10A, SCN11A, SCN5A, SETD2, SETD5, SGO1, SHQ1, SLC25A20, SLC25A26, SLC25A38, SLC38A3, SLC6A1, SLC6A20, SLC6A6, STT3B, SUMF1, SYN2, TDGF1, TGFBR2, THRB, TKT, TMEM43, TMIE, TNNC1, TOP2B, TRAIP, TRAK1, TREX1, TRIM71, TRNT1, TSEN2, TTC21A, UQCRC1, VHL, WNT5A, WNT7A, XPC, ZMYND10, ATG7, ATP2B2, BTD, CCDC174, COLQ, DAZL, FANCD2, GHRL, IQSEC1, MRPS25, NGLY1, PPARG, RAF1, RARB, RPL15, SATB1, SGO1, SLC6A1, SLC6A6, SYN2, THRB, TMEM43, TOP2B, TSEN2, VHL, WNT7A, XPC
22	Del 9p24.3-p22.3	17.7% (53/300)	Micrognathia (29), Low-set ears (25), Intellectual disability (22), Abnormal pinna morphology (17), Trigonocephaly (17)	Atrial septal defect (19), Ventricular septal defect (19), Patent ductus arteriosus (14), Abnormality of cardiovascular system morphology (12), Pulmonic stenosis (5)	0.3% (1/300)	Transposition of the great arteries (1)	DOCK8, KANK1, SMARCA2
23	Dup 9p22.3	11.6% (5/43)	Micrognathia (3), Brachydactyly (2), Downslanted palpebral fissures (2), Finger clinodactyly (2), Intellectual disability (2)	Abnormal heart morphology (1), Abnormality of the cardiovascular system (1), Mitral regurgitation (1), Patent ductus arteriosus (1), Tricuspid regurgitation (1)	0	/	/
24	Del 16q21-q22.1	17.9% (5/28)	Intellectual disability (4), Micrognathia (4), Hypotonia (3), Low-set ears (3), Microcephaly (3)	Aortic valve stenosis (2), Atrioventricular canal defect (2), Patent ductus arteriosus (2), Abnormality of cardiovascular system morphology (1), Pulmonic stenosis (1)	0	/	/
25	Del 17p11.2	13.4% (13/97)	Global developmental delay (6), Intellectual disability (5), Abnormal facial shape (3), Downturned corners of mouth (3), Myopia (3)	Tetralogy of Fallot (6), Patent ductus arteriosus (3), Abnormal heart morphology (2), Abnormal ventricular septum morphology (1), Abnormality of cardiovascular system morphology (1)	0	/	/
26	Del 3p22.2	50.0% (1/2)	Abnormal mandible morphology (1), Abnormal pinna morphology (1), Abnormality of the outer ear (1), Absent nipple (1), Craniosynostosis (1)	Transposition of the great arteries (1)	0	/	/
27	Dup 6p25.3-p22.2	10.2% (12/118)	Intellectual disability (4), Narrow mouth (4), Abnormal pinna morphology (3), Anteverted nares (3), Brachydactyly (3)	Abnormality of cardiovascular system morphology (3), Ventricular septal defect (3), Atrioventricular canal defect (2), Pulmonic stenosis (2), Atrial septal defect (1)	0	/	/
28	Del 15q26.3	17.1% (20/117)	Low-set ears (10), Intellectual disability (9), Small for gestational age (7), Abnormal pinna morphology (6), Hypertelorism (6)	Ventricular septal defect (10), Atrial septal defect (6), Patent ductus arteriosus (6), Abnormality of cardiovascular system morphology (2), Pulmonic stenosis (2)	0	/	/

^*^The percentage of syndromic and isolated CHD was summarized from DECIPHER database

^#^Blue font indicates differential genes present in isolated CHD from DECIPHER but absent in syndromic CHD from this study. Red font indicates differential genes present in syndromic CHD from this study but absent in isolated CHD from DECIPHER

### Candidate gene prioritization

Next, we asked whether genes in these pathogenic or likely pathogenic CNVs were implicated in the cardiac phenotypes of patients with syndromic CHD. Among 1249 candidate genes, VarElect, OVA, and AMELIE prioritized 253, 200, and 169, respectively (Additional file 7: Table S5). With a ToppGene threshold of p-value < 0.05 and a ToppNet interaction count of ≥ 20, 236 genes were prioritized (Additional file 7: Table S5). The pathway enrichment analysis on prioritized genes by the four tools is listed in Table S6 (Additional file 8). We also analyzed the interaction networks of genes prioritized by the four tools using STRING (Additional file 9: Fig. S2). The genes prioritized by the four tools were similar to have interactions. There were 38/253 (15%) isolated genes (no connection to other genes) in the VarElect set, 18/200 (9%) in the OVA set, 19/169 (11%) in the AMELIE set, and 33/236 (14%) in the ToppGene set. Furthermore, an overlapping analysis of prioritized genes from the four tools was employed (Fig. S3A). Sixteen genes, including *ACVR2B*, *B9D1*, *FLCN*, *AGO2*, *GLDC*, *MERTK*, *RHEB*, *NT5E*, *MPDZ*, *MNX1*, *SCN3B*, *THRB*, *TFAP2A*, *SUMF1*, *VHL*, and *TXNRD2*, were found overlapping the four tools. We analyzed the expression pattern of the sixteen overlapping prioritized genes during the heart development of mice. The primary time window of heart development in mice is day 7.5–13.5 of embryonic development (E7.5-E13.5) [32]. As shown in Fig. S3, the mRNA expression of *Acvr2b*, *Ago2*, *Mertk*, *Mpdz*, and *Vhl* remained high during E7-E14 and decreased after maturation. These results suggested that these genes may be involved in heart development.

## Discussion

### Principal findings

Syndromic CHDs are linked to chromosomal abnormalities [33], CNVs [34], single gene defects, and undetermined causes. In 2010, the ACMG regarded CMA as a first-tier diagnostic method for developmental disabilities [35]. Then, several studies investigated the diagnostic yield of CMA in syndromic CHD. However, the sample sizes were small, and the contribution of CNVs in syndromic CHD from the Chinese cohort is not yet discussed. We used two CMA platforms to identify pathogenic or likely pathogenic CNVs in 109 subjects with syndromic CHD from the Chinese population. Whether a CNV contributes to a phenotype is according to various factors, including how it is inherited, the content of the genes, the copy number duplication or deletion, the array platform, and if it exists in the general population. In order to discuss submicroscopic structural changes of chromosomes, we removed patients with aneuploidies and gross chromosomal aberrations, filtered common CNVs in the general population database (DGV), and finally identified 34 CNVs in 28 patients.

### Clinical characteristics in patients with previously reported syndromes

Five of the 28 patients presented previously reported syndromes. The 1.3 Mb 1p36.33 deletion in case 11 overlapped the distal critical region of 1p36. The related phenotypes of this distal region include anterior fontanel abnormalities, hypothyroidism, cleft palate, seizures, sensorineural hearing loss, congenital heart defects, and cardiomyopathy [36]. Case 11 presented ventricular septal defect (VSD) and mental retardation, commonly seen in patients with 1p36 distal region deletions. Frequent phenotypes of DiGeorge syndrome (22q11.2 deletion syndrome) include cardiovascular abnormalities, immunodeficiency, subtle but characteristic facial features, palatal abnormalities, endocrine abnormalities, gastrointestinal abnormalities, and genitourinary abnormalities [37]. With 22q11.21 deletion, case 32 manifested TOF, right aortic arch (RAA), athymism, and immunodeficiency, and case 66 exhibited VSD, abnormal facial features, and narrow glottis. Of these phenotypes, narrow glottis was less frequent in patients with DiGeorge syndrome. Miller–Dieker syndrome, or 17p13.3 deletion syndrome, is characterized by various dysmorphic features. Chen et al. summarized 29 cases with Miller–Dieker syndrome. They found that lissencephaly, corpus callosum dysgenesis/agenesis, and conotruncal heart defects were detected prenatally in 41% (12/29), 17% (5/29), and 14% (4/29) of the cases, respectively [38]. Several other studies have also observed lissencephaly, epilepsy, craniofacial dysmorphisms, and congenital anomalies in patients with Miller–Dieker syndrome [39]. In case 76 with 17p13.3 deletion, mental retardation and VSD were observed. However, central nervous system anomalies were not determined due to this patient's lack of magnetic resonance inspection. Cri du Chat syndrome (5p deletion) is characterized by the typical cry, severe mental and developmental retardation, and sensitive alterations. Less frequent characteristics, including cardiac, skeletal, genitourinary, metabolic, or immune abnormalities, may also be present [40]. In case 99 with 5p15.33-p15.31 deletion, we identified VSD, mental retardation, and motor retardation, matching the symptoms of patients with 5p deletion. Dysmorphism and visceral disorders (including congenital heart disease), neurocognitive impairment, and sleep–wake rhythm disorders are common phenotypes of Smith–Magenis syndrome (17p11.2 deletion) [41]. In this study, case 107 with 17p11.2 deletion presented VSD, developmental disorder of speech and language, and motor retardation. These phenotypes were within the phenotype spectrum of Smith–Magenis syndrome.

### CNV pathogenicity prediction

Several approaches have been developed to predict CNV pathogenicity, including SVScore [42] (based on single-nucleotide polymorphism pathogenicity scores within CNV intervals), ACMG guidelines [22] (based on individual opinions on a series of scoring items), haploinsufficiency score [43], etc. X-CNV is a newly developed “one-stop” estimation tool that integrates diverse public data of CNVs and outperforms the SVScore, AnnotSV [44], and ClassifyCNV [45]. Therefore, X-CNV is a comprehensive approach to providing the pathogenic annotations of CNVs. Apart from X-CNV, we also used DECIPHER and the OMIM database to predict the pathogenicity of the 34 CNVs. Considering these three predicting methods, we determined 29 pathogenic or likely pathogenic CNVs in 24 patients. Among these CNVs, only del 22q11.21 was discovered recurrent in cases 32 and 66, indicating a high degree of heterogeneity of CNVs in syndromic CHD.

### Diagnostic yield of CMA in syndromic CHD cohorts from different countries or ethnic backgrounds

We summarized the diagnostic yield of CMA in syndromic CHD cohorts from different countries or ethnic backgrounds (Table 4), and it varied from 10.3 to 67.3%. The difference in diagnostic yield may be associated with the populations included, the platforms used, and the criteria for pathogenic, likely pathogenic, or causal CNVs. In our study, the diagnostic yield of CMA was 23.1% (24/104), excluding 5 cases with aneuploidies and gross chromosomal aberrations. It is higher than 18.1% (47/260) in the BCH cohort but lower than 32.5% (66/203) in the BCM1 cohort. Then, recurrent CNVs were compared in our study and previously reported cohorts. Among the 11 reported cohorts summarized in Table 4, the causal CNVs of syndromic CHD in the BCH cohort were not listed. Thus, we compared the remaining 10 cohorts with ours to find recurrent CNVs (Additional file 10: Table S7). 31 recurrent CNVs were found among all cohorts, and the deletions of 22q11.21, 1p36.33, 17p13.3, 17p11.2, 17q25.3, 11q23.3-q25, 13q33.1-q34, and 5q35.3 were recurrent in our study and previously reported cohorts. The top two recurrent regions of all CNVs in our cohort and previously reported cohorts were 22q11 and 1p36 deletions, consistent with the EHRA/HRS/APHRS/LAHRS expert consensus statement [1]. Heterogeneous phenotypes of CHD and extracardiac malformations were observed in syndromic CHD from different countries and ethnicities. We summarized each study's top 3 cardiac and extracardiac malformations (Table 3). In patients carrying pathogenic or likely pathogenic CNVs from Greece, Brazil, China, and the Caucasian population, simple CHD, such as septal defects, was most common. In two studies that included patients from Belgium, we found that isolated conotruncal and septal defects were the most frequent cardiac phenotypes. Furthermore, neurodevelopmental disorders were the most common extracardiac comorbidities of patients from Greece and China. Craniofacial defects were the most frequent extracardiac comorbidities in cases from Belgium, Brazil, and the Caucasian population.

Of all CNVs non-recurrent between our cohort and previously reported cohorts, 3q25.33-q26.1 deletion (case 17), 8q24.21-q24.3 duplication (case 5), and 3p26.3-p24.2 duplication (case 102) were not published previously. Case 17 presented double outlet right ventricle (DORV), transposition of the great arteries (TGA), VSD, pulmonic stenosis (PS), PDA, abnormal right coronary artery branch, hydrocele, and genu valgum. Chang et al. [46] identified 3q25 deletion in 12 patients. They found that the CNV was associated with developmental delay, microcephaly, synophrys, epicanthus, ptosis, blepharophimosis, broad nasal bridge, ear abnormalities, and cardiac defects. Among these phenotypes, cardiac defects overlapped between patients with 3q25 deletion and case 17 with 3q25.33-q26.1 deletion. Case 5 carried two pathogenic CNVs, 13q33.1-q34 deletion, and 8q24.21-q24.3 duplication. He et al. discovered that patients carrying 13q33-q34 deletions had a high risk of developmental disability, facial deformity, CHD, and other malformations [47]. 8q24.21 is a hot spot associated with cancer, but the relationship between 8q24.21-q24.3 and CHD or other congenital malformations is not discussed yet. The phenotypes of case 102 were VSD, atrial septal defect (ASD), hypothyroidism, and developmental delay. Previous studies have discovered 3p26.3 microduplication in some patients with non-syndromic intellectual disability [48, 49]. Their CNV lengths were shorter than case 102, indicating that the inconsistent phenotypes of 3p26.3 duplication may be attributed to different lengths of CNV intervals.

### Discovering novel CHD candidate genes by CNV detection

Previous studies have demonstrated that the number of candidate genes of different prioritization tools varied significantly. Qiao et al. used five prioritization web tools to identify candidate genes of subjects with intellectual disabilities and found a discrepancy in candidate gene sets of different web tools [50]. Jayaraman et al. used the software ENDEAVOUR, ToppGene, and DIR to rank candidate genes of leukemogenesis [51]. They found that the top 100 ranked genes from each tool differed, and only 54 genes overlapped in priority gene lists from these prediction approaches. As prioritization web tools using various databases and algorithms, many recent studies have recommended combining multiple web tools to identify critical candidate genes [52–54]. In this study, we used four gene prioritization tools to prioritize candidate genes of CHD within pathogenic or likely pathogenic CNVs. Our data also showed discrepancies in different priority lists (Additional file 11: Fig. S3A). The pathway enrichment analysis showed that the priority lists were enriched in different pathways associated with heart development. Thus, the combination of multiple web tools is necessary to identify phenotype-related genes and find critical candidate genes comprehensively. The overlapping analysis between priority lists suggested 16 genes as candidate genes associated with CHD. Furthermore, 31.3% (5/16) of the overlapping prioritized genes between four tools showed a high mRNA expression during the critical time window of heart development in mice. Cardiac phenotypes were observed in the targeted homozygous null allele mice of 87.5% (14/16) of the prioritized genes according to the MGI database, indicating that the prioritization process can highlight CHD-related genes. Of note, mice homozygotes for the targeted null alleles of *Acvr2b*, *B9d1*, and *Gldc* exhibit septal defects, which can be observed in the corresponding patients.

The sixteen prioritized genes were associated with eleven cases, and four carried abnormal copy numbers of at least two prioritized genes (cases 77, 102, 103, and 107). Previous studies have discovered that genetic disturbance in CHD is a multi-factorial, polygenic etiology [55, 56]. Single-nucleotide variants analyses in patients with CHD have also demonstrated that oligogenic or polygenic variants may contribute together to the pathogenesis of CHD [57, 58]. As there are dosage alterations of multiple genes in each CNV, it highlights efforts to understand the roles of multiple genes in the phenotypes. Morrow et al. summarized the molecular genetics of 22q11.2 deletion syndrome and highlighted the combined roles of the loss of *TBX1*, *CRKL*, and *DGCR8* in 22q11.2-caused congenital malformations. Other genes mapped to this region, such as *COMT*, *PRODH*, and *PIK4CA*, may contribute to cognitive and behavioral problems in patients with 22q11.2 deletion [59]. In this study, case 102 carried duplication of *VHL*, *SUMF1*, and *THRB*, which were prioritized. Other genes, including *CAV3*, *COLQ*, *CRELD1*, *RAB5A*, *RAF1*, *RARB*, *SLC6A6*, *CRBN*, *PPARG*, and *WNT7A*, were also associated with cardiovascular system phenotypes according to the MGI database. Although the prioritization process identified several CHD-related genes, the consideration of the possibility that multiple genes on each CNV may contribute to the phenotypes together is needed. Further model organism research should focus on this issue and comprehensively uncover the polygenic etiology of syndromic CHD.

Another issue is that certain ethnic or racial groups tend to have more CHD-susceptible variants and influence the prevalence and outcomes of CHD [60]. For example, a meta-analysis revealed that MTHFR gene 677 T polymorphism was a genetic risk factor in the development of CHD in the Chinese population [61]. Lahm et al. [62] also identified multiple risk loci for all major CHD subgroups in patients of German ethnicity. In this study, we detected several CNVs from the Chinese population and provided a unique source for identifying novel CHD candidate genes. For each CNV, we listed CHD-related genes for the reference of future functional studies. And the sixteen overlapping genes are considered to be the most likely candidate CHD genes.

### Strengths and limitations

Our study focused on patients with syndromic CHD in the Chinese population, which enabled us to discuss the role of CNVs in both CHD and multiple extracardiac abnormalities. However, there are some limitations in our study. Firstly, we only included sporadic cases, and the parents of all cases were not included. Secondly, as the extracardiac phenotypes were variable in our study, finding the relationship between CNVs and a specific extracardiac phenotype was not easy. Therefore, we only described the phenotype spectrum of each pathogenic or likely pathogenic CNV in syndromic CHD from DECIPHER database and this study. Moreover, the gene prioritization process was only performed for CHD-related genes. In the future, syndromic CHD involving a specific subtype of extracardiac malformations with larger sample size is needed further to delineate the correlation between CNV and syndromic CHD.

## Conclusions

This study firstly applied CMA and bioinformatic analysis to explore syndromic CHD-related CNVs and genes from the perspective of the Chinese population. The pathogenic or likely pathogenic CNVs found in this study extended our understanding of the chromosomal aberrations in syndromic CHD. The combination of prioritization tools was essential in prioritizing CHD candidate genes and helping discover the pathogenesis of syndromic CHD.

## Supplementary Information

**Additional file 1**. **Table S1.** Summary of tools for candidate gene prioritization, including VarElect, OVA, AMELIE, and ToppGene.

**Additional file 2**. **Table S2.** Training gene set generated from RDDC, Phenopedia, and DisGeNET.

**Additional file 3**. **Additional methods.** Pathway analysis of the prioritized genes, the databases, and the dataset.

**Additional file 4**. **Table S3.** The remaining 85 patients with syndromic CHD.

**Additional file 5**. **Fig. S1.** Gene ontology analysis of the 1249 candidate genes (1129 matched).

**Additional file 6**. **Table S4.** CNVs in studies of syndromic CHD from different countries or ethnicities.

**Additional file 7**. **Table S5.** Prioritized genes of four tools.

**Additional file 8**. **Table S6.** Pathway enrichment analysis on prioritized genes by different tools.

**Additional file 9**. **Fig. S2.** Interaction analyses of prioritized genes by different tools.

**Additional file 10**. **Table S7.** Recurrent CNVs in previously published syndromic CHD cohorts and our study.

**Additional file 11**. **Fig. S3.** The prioritized genes overlapping between the four tools.

## Data Availability

The datasets are available from the corresponding author upon request.

## Abbreviations

CHD: Congenital heart disease
CNV: Copy number variant
CMA: Chromosomal microarray analysis
MVP: Meta-voting prediction
STRING: Search Tool for Retrieval of Interacting Proteins
MGI: Mouse genome informatics
ASD: Atrial septal defect
VSD: Ventricular septal defect
AVSD: Atrioventricular septal defect
TR: Tricuspid regurgitation
MR: Mitral regurgitation
PS: Pulmonic stenosis
MS: Mitral stenosis
AS: Aortic stenosis
CoA: Coarctation of the aorta
TOF: Tetralogy of Fallot
DORV: Double outlet right ventricle
TGA: Transposition of the great arteries
PA: Pulmonary atresia
PAPVC: Partial anomalous pulmonary venous connection
PAS: Pulmonary artery sling
SV: Single ventricle
PDA: Patent ductus arteriosus
F: Female
M: Male
NM: Not mentioned
RAA: Right aortic arch
PVS: Pulmonary valve stenosis
