# Supplementary material for: Copy number variant analysis for syndromic congenital heart disease in the Chinese population
Source: Hum Genomics. 2022 Oct 31;16:51. doi: 10.1186/s40246-022-00426-8 (PMC9623925; doi:10.1186/s40246-022-00426-8)
Supplement: Supplementary file 3 — Additional file 3. Additional methods. Pathway analysis of the prioritized genes, the databases, and the dataset. [file 40246_2022_426_MOESM3_ESM.docx]

**Additional methods**

**Pathway analysis of the prioritized genes**

Three web tools were used for pathway analysis of the prioritized genes, including: DAVID Bioinformatics Resources (<https://david.ncifcrf.gov/>) [1], WEB-based GEne SeT AnaLysis Toolkit (WebGestalt, <http://www.webgestalt.org/>) [2], and Metascape(<https://metascape.org/gp/index.html>) [3].

**Other databases and dataset**

The interaction networks of the genes prioritized by different tools were constructed by the Search Tool for Retrieval of Interacting Proteins [4] (STRING, <https://cn.string-db.org/).> The Mouse Genome Informatics (MGI) database [5] (http://www.informatics.jax.org/) identifies whether cardiovascular system phenotypes were present in the mice with targeted homozygous null alleles of prioritized genes. The dataset GSE51483 [6] contains the expression profile of murine cardiogenesis from the embryonic stage to the adult stage. Therefore, the expression profile of the overlapping prioritized genes during murine cardiogenesis was extracted from the dataset GSE51483.

**References**

1. Sherman BT, Hao M, Qiu J, Jiao X, Baseler MW, Lane HC, et al. DAVID: a web server for functional enrichment analysis and functional annotation of gene lists (2021 update). Nucleic acids research. 2022.

2. Liao Y, Wang J, Jaehnig EJ, Shi Z, Zhang B. WebGestalt 2019: gene set analysis toolkit with revamped UIs and APIs. Nucleic acids research. 2019;47(W1):W199-w205.

3. Zhou Y, Zhou B, Pache L, Chang M, Khodabakhshi AH, Tanaseichuk O, et al. Metascape provides a biologist-oriented resource for the analysis of systems-level datasets. Nature communications. 2019;10(1):1523.

4. Szklarczyk D, Gable AL, Nastou KC, Lyon D, Kirsch R, Pyysalo S, et al. The STRING database in 2021: customizable protein-protein networks, and functional characterization of user-uploaded gene/measurement sets. Nucleic acids research. 2021;49(D1):D605-d12.

5. Bult CJ, Blake JA, Smith CL, Kadin JA, Richardson JE. Mouse Genome Database (MGD) 2019. Nucleic acids research. 2019;47(D1):D801-d6.

6. Li X, Martinez-Fernandez A, Hartjes KA, Kocher JP, Olson TM, Terzic A, et al. Transcriptional atlas of cardiogenesis maps congenital heart disease interactome. Physiological genomics. 2014;46(13):482-95.
