# Supplementary figures and images for: Copy number variant analysis for syndromic congenital heart disease in the Chinese population
Source: Hum Genomics. 2022 Oct 31;16:51. doi: 10.1186/s40246-022-00426-8 (PMC9623925; doi:10.1186/s40246-022-00426-8)

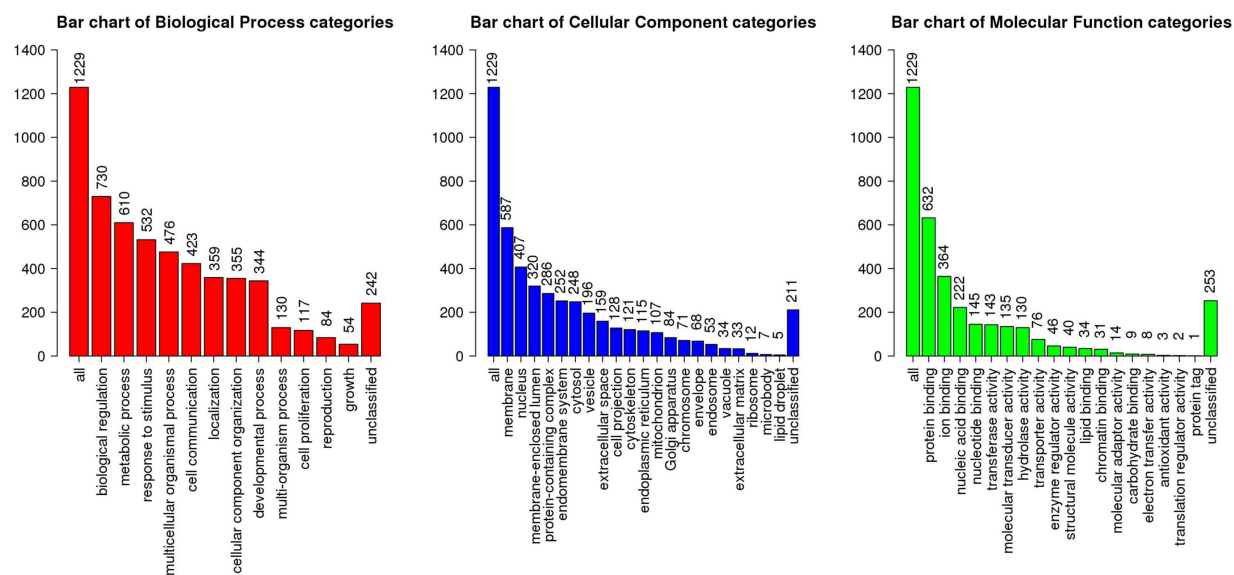

Fig. S1 Gene Ontology analysis of the 1249 candidate genes (1229 matched)

Supplement: Supplementary file 5 — Additional file 5. Fig. S1. Gene ontology analysis of the 1249 candidate genes (1129 matched). [file 40246_2022_426_MOESM5_ESM.pdf]
