# Supplementary material for: Copy number variant analysis for syndromic congenital heart disease in the Chinese population
Source: Hum Genomics. 2022 Oct 31;16:51. doi: 10.1186/s40246-022-00426-8 (PMC9623925; doi:10.1186/s40246-022-00426-8)
Supplement: Supplementary file 11 — Additional file 11. Fig. S3. The prioritized genes overlapping between the four tools. [file 40246_2022_426_MOESM11_ESM.pdf]

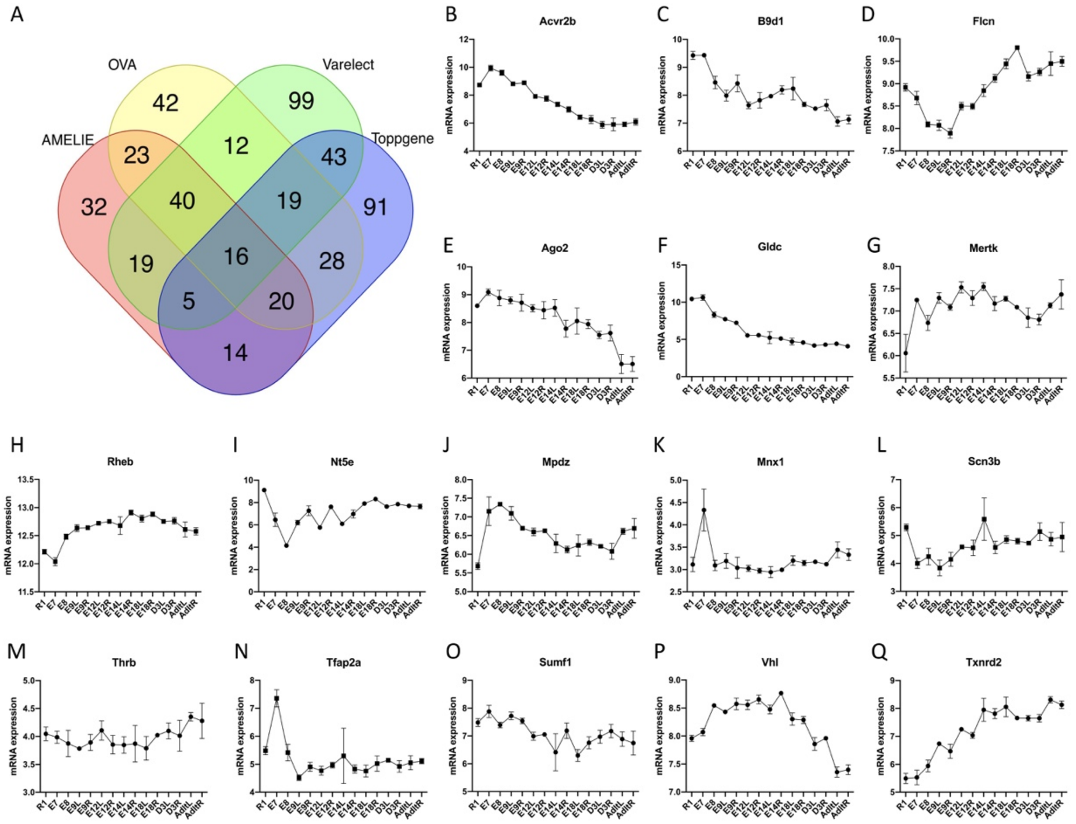

Fig. S3 The prioritized genes overlapping between the four tools. (A) Venn diagram representing the number of genes identified with each of the four tools and their overlaps; (B-Q) Normalized mRNA expression of the sixteen overlapping prioritized genes during heart development in mice (GSE51483).
