# Supplementary material for: Copy number variant analysis for syndromic congenital heart disease in the Chinese population
Source: Hum Genomics. 2022 Oct 31;16:51. doi: 10.1186/s40246-022-00426-8 (PMC9623925; doi:10.1186/s40246-022-00426-8)
Supplement: Supplementary file 9 — Additional file 9. Fig. S2. Interaction analyses of prioritized genes by different tools. [file 40246_2022_426_MOESM9_ESM.pdf]

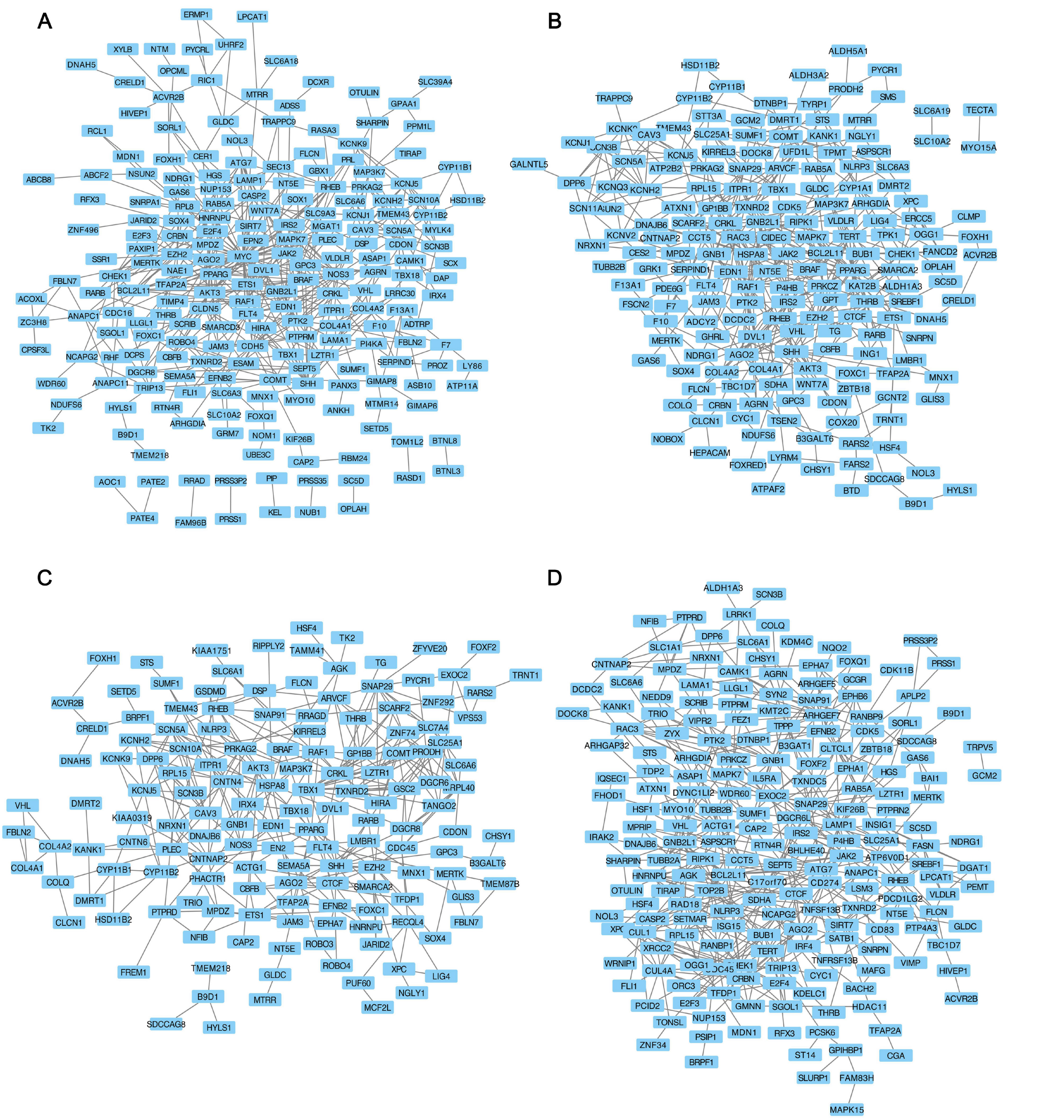

Fig. S2 Interaction analyses of prioritized genes by different tools. (A) VarElect; (B) OVA; (C) AMELIE; (D) ToppGene.
